# Reactivity to Smoking Cues in a Social Context: Virtual Reality Experiment

**DOI:** 10.2196/71285

**Published:** 2025-05-26

**Authors:** Katharina Eidenmueller, Sabine Hoffmann, Kornelius Kammler-Sücker, Leonard Wenger, Massimiliano Mazza, Christiane Mühle, Manuel Stenger, Gerrit Meixner, Falk Kiefer, Bernd Lenz

**Affiliations:** 1 Partner Site Mannheim-Heidelberg-Ulm, Deutsches Zentrum für Psychische Gesundheit (German Center for Mental Health), Mannheim, Germany; 2 Department of Addictive Behavior and Addiction Medicine, Central Institute of Mental Health, J5, Mannheim, 68159, Germany, 49 62117033827; 3 Center for Innovative Psychiatric and Psychotherapeutic Research (CIPP), Virtual Reality Lab, Central Institute of Mental Health, Mannheim, Germany; 4 Department of Psychiatry and Psychotherapy, Friedrich-Alexander University Erlangen-Nürnberg (FAU), Erlangen, Germany; 5 UniTyLab, Faculty of Informatics, Heilbronn University, Heilbronn, Germany

**Keywords:** virtual reality, smoking, cue reactivity, craving, social context

## Abstract

**Background:**

Social contextual factors influence the onset and maintenance of substance abuse. Virtual reality (VR) provides a standardized method to present social stimuli and is increasingly used in addiction research.

**Objective:**

This study examines the influence of a smoking versus a nonsmoking agent in VR on craving in nicotine-dependent male participants. Our primary hypothesis was that the interaction with a smoking agent is associated with increased craving compared to a nonsmoking agent. We expected higher craving in the presence of an agent regardless of the agent’s smoking status.

**Methods:**

Using a head-mounted display (Oculus Rift), 50 nicotine-dependent smokers were exposed to four VR conditions on a virtual marketplace: first without an agent, second and third with an agent who either smoked or did not smoke in randomized order, and fourth without an agent as a follow-up condition. Before the follow-up condition, participants smoked a cigarette. Craving was assessed with the Questionnaire of Smoking Urges and a visual analog scale within VR and after each session. We also examined anxiety and agitation (visual analog scale), immersion and presence with the igroup Presence Questionnaire, and salivary cortisol levels.

**Results:**

Results showed no significant difference in the participants’ craving, anxiety, or agitation between the smoking and nonsmoking agent conditions. However, craving, anxiety, and agitation increased from the marketplace without an interacting agent to the conditions with an interacting agent, and decreased after smoking a cigarette. Immersion was low in all conditions and decreased over time. Salivary cortisol levels were highest at baseline and decreased over the course of the experiment.

**Conclusions:**

These findings suggest that the presence of an agent (as a contextual factor) may override the specific influence of proximal stimuli (burning cigarette). The low immersion highlights the challenges in developing effective VR environments for cue exposure.

## Introduction

Despite its decreasing prevalence, cigarette smoking remains a global health challenge. According to the latest report of the Global Burden of Disease study from 2019, a total of 1.24 billion people were current smokers, and 1.69 million deaths occurred as a consequence of tobacco use globally [1]. One of the diagnostic criteria for tobacco use disorder is craving [2], which describes the strong urge or desire to smoke. Craving is a well-established driver of smoking behavior [3,4] and relapse after smoking cessation [5].

A meta-analysis on cue-reactivity in smokers shows that the presentation of smoking-related cues can induce craving for cigarettes and that self-reported craving is a valuable index for cue-reactivity, as effect sizes were robust across different moderators. In contrast, the effects of physiological measures of cue reactivity remained small or nonsignificant [6]. Social context was found to modulate cue-induced craving [7,8]. Dimoff and Sayette [9] make a plea for including social contextual factors in laboratory experiments on smoking in order to gain a deeper understanding of the mechanisms underlying smoking behavior and craving. The authors point out that only a few studies examine the influence of social context on smoking behavior, but those that do suggest an influence of the presence of others on smoking behavior, the effects of smoking, and self-regulatory and perceptive processes related to smoking. Adolescents with peers who are smokers are more likely to smoke or to start smoking [10]. Field studies using ecological momentary assessment found that smokers are particularly likely to smoke when socializing [11] or in the presence of other people who are smoking [12,13]. The presence of people who are not smoking, on the other hand, appears to suppress smoking behavior [14]. Social context was found to enhance cue-induced craving compared to cues presented in a neutral context in one study [7], whereas it seemed to reduce cue-induced craving for cigarettes in a study using virtual reality (VR) [8]. Which specific processes are involved in the modulation of smoking behavior and cue-induced craving in a social context has yet to be determined.

Stress hormones like cortisol take effect in the context of social stress, and increased cortisol levels in response to social stressors have been shown to be associated with increased craving in smokers [15,16] and increased reactivity to smoking cues [17]. This could indicate cortisol levels as a potential mediator between social context and cue-induced craving.

VR is a promising tool for studying social context, as it facilitates the standardized and controlled presentation of complex social situations in experimental designs without sacrificing ecological validity [18,19]. Moreover, VR simulations have been successfully used to induce cigarette craving [20] and the accompanying physiological reactions [21] in addicted smokers. Smoking cues in VR appear to induce stronger cravings than the presentation of smoking-related images [22]. Different VR environments can induce cigarette craving (bar, restaurant, street, and home scenarios) [23]. Virtual party scenarios that include social interactions with virtual agents who smoke and offer cigarettes induce higher levels of craving than a virtual paraphernalia room with smoking cues [20]. Furthermore, VR-based cue exposure therapy has been successfully implemented for smoking cessation [24], but there are also null findings of VR cue exposure on self-reported craving [25]. The subjective level of presence in the VR is positively correlated with craving for cigarettes [26]. As VR has been shown to be an appropriate tool for studying social context, as well as for the induction of craving in smokers, the use of VR appears to be a suitable approach for examining the influence of social contextual factors on craving for cigarettes.

In this study, our primary aim was to examine the influence of the interaction with a smoking versus a nonsmoking virtual agent on craving and stress hormone levels in smokers with tobacco use disorder. Study participants were immersed in a VR setting in which they interacted with a virtual agent who was, depending on the condition, either smoking a cigarette or not. Craving, anxiety, and agitation were then assessed within the VR, and saliva samples were collected after each trial. We hypothesized that the presence of a smoking agent would be associated with an increase in craving compared to the nonsmoking agent. We also expected a difference in craving levels in the presence of an agent compared to the absence of an agent, regardless of smoking status, and a decrease in craving after smoking a cigarette. Moreover, we explored the effects on anxiety and agitation and the association between salivary stress hormone levels and cue-elicited craving in VR.

## Methods

### Participants

The study sample consisted of 50 male smokers aged between 18 and 64 (mean 30.38, SD 10.97) years. Sample characteristics are depicted in Table 1. Only smokers who met the Diagnostic and Statistical Manual of Mental Disorders, 5th edition criteria for tobacco use disorder were included. Because the menstrual cycle has been associated with substance use behaviors [27] and controlling for the phases would have required a large number of participants, no female participants were recruited for this pilot study. Further exclusion criteria were psychiatric comorbidities, having had more than one psychiatric or psychotherapeutic treatment session within 10 years prior to recruitment, current intake of psychoactive medication, severe somatic disorders or neurological disorders, epilepsy, and illegal drug use. Participants who had ametropia of more than three diopters and could not wear contact lenses were also excluded to ensure a clear vision of the VR scenario. Participants were questioned about these criteria in a telephone screening before being included in the study.

**Table 1.** Demographic and clinical characteristics of study participants (N=50).

	12-hour abstinence (n=20)	30-minute abstinence (n=30)	*t* test (df)	*P* value
Age (years), mean (SD)	28.05 (10.39)	31.93 (11.24)	–1.23 (48)	.22
Severity of nicotine dependence (according to the FTND^a^ sum score)^b^, %				.18
Low	10	3.3	N/A^c^	N/A
Moderate	45	33.3	N/A	N/A
High	30	40	N/A	N/A
Very high	15	23.3	N/A	N/A
FTND sum score, mean (SD)	5.0 (2.18)	5.97 (1.65)	–1.79 (48)	.08
Age at first cigarette (years), mean (SD)	15.10 (3.64)	14.93 (2.49)	1.93 (48)	.85
Cigarettes per day, mean (SD)	12.20 (7.44)	12.87 (5.69)	–0.36 (48)	.72
Years of smoking, mean (SD)	12.05 (11.70)	14.73 (9.94)	–0.87 (48)	.39
PSS^d^ score, mean (SD)	15.10 (5.48)	13.74 (6.01)	0.80 (48)	.43

a Fagerström Test for Nicotine Dependence.

b For nicotine dependence*: U*=41.38; *Z*=–1.33.

c N/A: not applicable.

d Perceived Stress Scale.

Of the 50 participants, the first 20 were asked to remain abstinent from smoking for 12 hours before the study appointment. Adherence to this was checked with a carbon monoxide breath test. The remaining 30 participants were instructed to abstain from smoking for 30 minutes prior to the study appointment. The two groups did not differ significantly in age, number of cigarettes per day, age of first cigarette, smoking years, and severity of nicotine dependence (Table 1).

### Ethical Considerations

Study participation was voluntary, and all participants provided written informed consent prior to participation. The study was carried out in accordance with the Declaration of Helsinki and was approved by the Ethics Committee II of the Medical Faculty Mannheim, Heidelberg University, Germany (#2021-504). Participants received a compensation of 50€ (US$ 56.23) for study participation. The data was anonymized.

### Measures

The severity of nicotine dependence was assessed with the Fagerström Test for Nicotine Dependence [28]. The Perceived Stress Scale [29] was used to assess participants’ self-reported levels of stress. Cigarette craving was assessed with the Questionnaire of Smoking Urges (QSU). The QSU has two subscales, which represent the expected positive consequences of smoking (factor 1) and relief from unpleasant states (factor 2) [30]. The 7-point visual analog scales (VAS) were used to assess craving, anxiety, and agitation during the VR trials. To measure the level of immersion in the virtual environment, the igroup Presence Questionnaire (iPQ) was used, assessing participants’ sense of realism, spatial presence, and involvement in the VR in distinct scales [31]. As VR simulations have the potential to induce motion sickness in participants, the Simulator Sickness Questionnaire [32] was used to be able to control symptoms of motion sickness. Saliva samples were analyzed for cortisol levels by ELISA (RE52611, IBL International GmbH, Hamburg, Germany).

### Virtual Environment and Apparatus

The virtual environment was presented on a head-mounted display (Oculus Rift, Reality Labs/Meta Platforms, Menlo Park, California), together with the accompanying Touch Controllers for user interaction. The experiment was programmed using the Python-based software Vizard 6 (WorldViz). The 3D environment was a customized own design, based on templates provided with Vizard and other models purchased from various sources and manually adapted with 3d Studio (Autodesk). The virtual companion character was created with Fuse CC beta (Adobe Inc) and rigged and animated with Mixamo (Adobe Inc), with its own adaptations to the animation to include smoking movements (created with MotionBuilder, Autodesk). We used voice recordings of a volunteer for the character’s dialogue parts when chatting with the participant.

**Figure 1.**
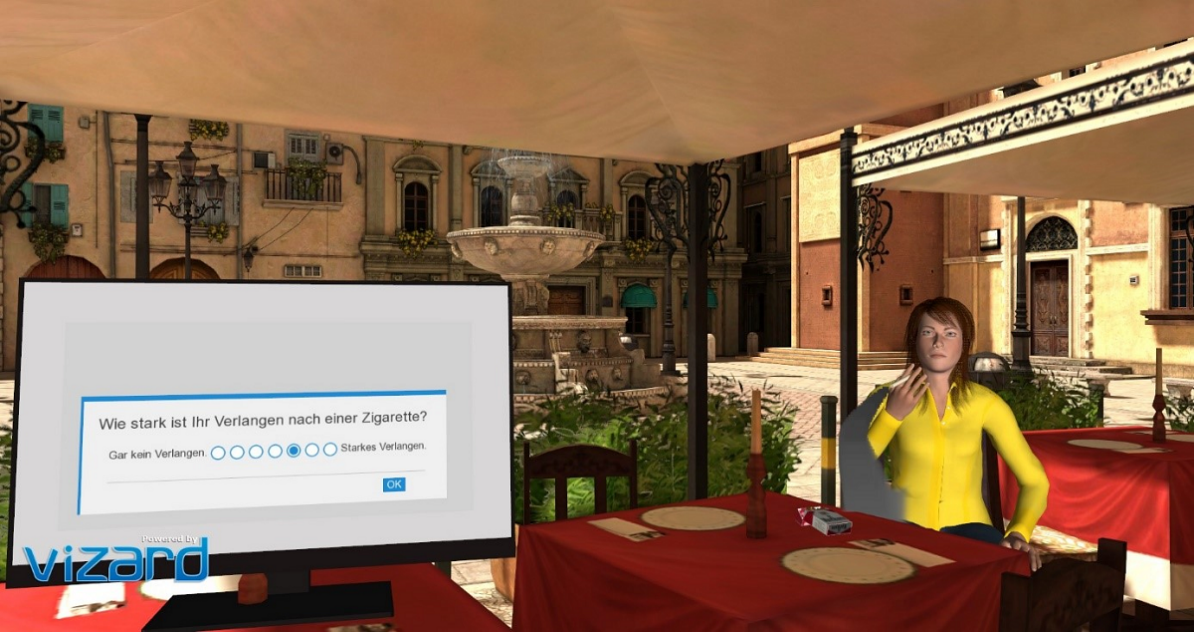
Screenshot of the market square in the smoking agent condition. On the computer screen within the VR, the craving visual analog scale is depicted (“How strong is your craving for a cigarette?” in German language). VR: virtual reality.

Within the virtual environment, participants found themselves seated under a sunshade in an outdoor café on a plaza of roughly Mediterranean style. In the distance, two virtual characters sat on a bench, chatting with each other, and a fountain on the square would provide a constantly burbling background sound. Besides these aspects, the further environment was free of distractions and designed to provide a comfortable atmosphere. Placed on a table directly in front of the participants, they found a virtual computer screen, which displayed all the following instructions and visual reaction tasks. When in either one of the agent conditions, the virtual character faced the participants while leisurely sitting on a neighboring table to their right (Figure 1). The character then involved the participants in a short dialogue, which included the character commenting on how stressed out the participant appeared (both agent conditions), and offered a cigarette two times (smoking agent condition). During the dialogue, participants were asked via screen instructions to respond, and then to indicate that they had finished with a button press on the handheld controller. After the dialogue, the virtual character remained seated, either comfortably idling (nonsmoking condition) or taking a drag from her smoking cigarette from time to time (smoking condition). The smoking animation was repeated with nonregular (randomized), yet high frequency, with the character dragging or puffing out for roughly 47% of the time. The smoking animation included an appreciative inhalation sound and a prolonged puffing out of virtual smoke with accompanying sound effects. In the smoking agent condition, this continued throughout all tasks.

### Procedure

**Figure 2.**
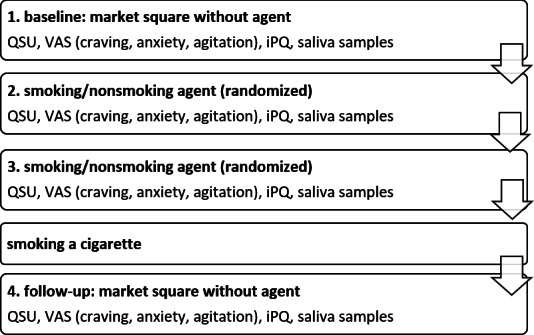
Flowchart of the experimental procedure. iPQ: igroup Presence Questionnaire; QSU: Questionnaire of Smoking Urges; VAS: visual analog scale.

Prior to the assessment, a telephone screening was conducted to ensure participants met the diagnostic criteria for tobacco use disorder and to check for exclusion criteria. At the study appointment, a carbon monoxide breath test was conducted on arrival for the participants who were asked to remain abstinent from smoking for 12 hours prior to the assessment. At the beginning of the appointment, all participants were asked to fill in an initial set of questionnaires on a laptop (sociodemographic information, Fagerström Test for Nicotine Dependence, and Perceived Stress Scale). Subsequently, four VR trials were conducted. For the baseline assessment, participants were presented with the virtual marketplace with no agent present. The second and third were the trials that included the interaction with the virtual agent, who was either smoking and offering cigarettes, or not smoking and asking participants about daily stressors. The order in which these conditions were presented was randomized. In the fourth trial, participants were once again presented with the empty marketplace with no agent present as a follow-up measurement. In each trial, after interacting with the agent or after getting some time to familiarize themselves with the marketplace (for trials with no agent present), the VAS were conducted on the computer screen within the VR. Each trial took about 20 minutes. After each VR trial, participants filled in a set of questionnaires on a laptop (QSU, iPQ, and Simulator Sickness Questionnaire) and provided saliva samples, which took around 10 minutes each time. Before the follow-up trial, participants smoked a cigarette during a 10-minute cigarette break. Figure 2 shows a flowchart of the experimental procedure.

### Statistical Analysis

In order to compare the smoking with the nonsmoking agent conditions, linear mixed models were fitted for both QSU scales, VAS (craving, anxiety, and agitation), iPQ total score, and cortisol as dependent variables with age, duration of prior abstinence, sequence (smoking or nonsmoking agent first), time point (trials 2 or 3), and condition (smoking vs nonsmoking agent) as predictors. Cortisol levels were logarithmized prior to the analysis. To analyze changes over the course of the experiment, multilevel models were fitted with both the QSU scale, VAS (craving, anxiety, and agitation), cortisol, and the iPQ total score as the dependent variables and age, duration of prior abstinence, sequence (smoking or nonsmoking agent first), and condition (baseline (T0), smoking|nonsmoking (T1|T2), follow-up (T3)) as predictors. We estimated multilevel (mixed) models with random intercepts to account for the clustered structure of the data points within participants (level 1: VR condition; level 2: participant). We report *F* tests for main effects in the generalized linear mixed models, followed by 2-tailed *t* tests for the parameter estimates β, indicating differences from the reference category in categorical variables; *t* values are shown for predictors with three or more categories. Statistical analyses were conducted using SPSS Statistics version 29; IBM Corp) for Windows. Results were regarded as significant when the 2-sided *P* value was below .05.

## Results

### Differential Effects Between Smoking and Nonsmoking Agents

There was no significant difference between the effects of the smoking agent compared to the effects of the nonsmoking agent on explicit craving in both QSU scales and the VAS (Table 2). For the QSU factor 2 subscale and the VAS, the craving was higher in the group with longer abstinence, and for the craving VAS, the time point (trials 2 or 3) was a significant predictor, with higher craving for the third trial. The effects shown also remain when the duration of abstinence is taken into account. The duration of abstinence increases craving but is not a moderator for the effects of the VR in our models (Table S1 in Multimedia Appendix 1).

**Table 2.** Results of a multilevel model with craving as the dependent variable and age, prior abstinence, sequence, time point (trials 2 or 3), and condition as predictors.

	β (95% CI)		*F* test (*df*)		*P* value
QSU^a^ factor 1 (cases n=50; observations n=93)					
Age	.196 (–0.138 to 0.529)		0.514 (1, 45)		.48
Prior abstinence	—^b^		5.548 (1, 45)		.24
12 hours	8.654 (1.259 to 16.049)		—		—
30 minutes	[reference]				
Sequence	—		0.045 (1, 46)		.83
Smoking agent first	–.759 (–7.975 to 6.456)		—		—
Nonsmoking agent first	[reference]				
Time point	—		0.883 (1, 42)		.35
Second trial	–1.039 (–3.273 to 1.194)		—		—
Third trial	[reference]				
Condition	—		0.131 (1, 42)		.72
Smoking Agent	–.401 (–2.635 to 1.833)		—		—
Nonsmoking Agent	[reference]				
QSU factor 2 (cases n=50; observations n=99)					
Age	.155 (–0.235 to 0.545)		0.641 (1, 46)		.43
Prior abstinence	—		5.792 (1, 46)		.02^c^
12 hours	10.304 (1.685 to 18.922)		—		—
30 minutes	[reference]				
Sequence	—		0.338 (1, 46)		.56
Smoking agent first	2.431 (–5.993 to 10.856)		—		—
Nonsmoking Agent First	[reference]				
Time point	—		0.970(1, 47)		.33
Second trial	–.861 (–2.620 to 0.898)		—		—
Third trial	[reference]				
Condition	—		1.422 (1, 47)		.24
Smoking agent	–1.043 (–2.802 to 0.716)		—		—
Nonsmoking agent	[reference]				
Craving VAS^d^ (cases n=50; observations n=100)					
Age	–.008 (–0.044 to 0.028)		0.190 (1, 46)		.66
Prior abstinence	—		6.699 (1, 46)		.01^c^
12 hours	1.017 (0.226 to 1.809)		—		—
30 minutes	[reference]				
Sequence	—		0.308 (1, 46)		.58
Smoking Agent First	–.213 (–0.987 to 0.560)		—		—
Nonsmoking agent first	[reference]				
Time point	—		4.384 (1, 48)		.04^c^
Second trial	–.451 (–0.885 to 0.018)		—		—
Third trial	[reference]				
Condition	—		0.191 (1, 48)		.66
Smoking agent	.094 [–0.339 to 0.528)		—		—
Nonsmoking agent	[reference]				

a QSU: Questionnaire of Smoking Urges.

b Not applicable.

c *P*<.05.

d VAS: visual analog scale.

There were also no significant differential effects of the smoking versus the nonsmoking agent on the anxiety or agitation VAS, the IPQ score (presence and immersion), or the saliva cortisol concentrations (Table 3). There was a significant increase in cortisol concentrations from the second to the third trial.

**Table 3.** Results of a multilevel model with anxiety, agitation, presence, and cortisol as the dependent variable and age, prior abstinence, sequence, time point (trials 2 or 3), and condition as predictors^a^.

	β (95% CI)		*F* test (df)		*P* value
Anxiety VAS^b^					
Age	.002 (–0.027 to 0.031)		0.013 (1, 46)		.91
Prior abstinence	—^c^		1.454 (1, 46)		.23
12 hours	–.385 (–1.027 to 0.258)		—		—
30 minutes	[reference]				
Sequence	—		0.694 (1, 46)		.41
Smoking agent first	–.260 (–0.888 to 0.368)		—		—
Nonsmoking agent first	[reference]				
Time point	—		0.176 (1, 46)		.68
Second trial	.060 (–0.228 to 0.348)		—		—
Third trial	[reference]				
Condition	—		1.362 (1, 46)		.25
Smoking agent	–.167 (–0.455 to 0.121)		—		—
Nonsmoking agent	[reference]				
Agitation VAS					
Age	–.30 (−0.067 to 0.006)		2.746 (1, 46)		.10
Prior abstinence	—		0.588 (1, 46)		.45
12 hours	–.308 (−0.501 to 1.117)		—		—
30 minutes	[reference]				
Sequence	—		0.411 (1, 46)		.53
Smoking agent first	–.252 (–1.04 to 0.539)		—		—
Nonsmoking agent first	[reference]				
Time point	—		0.043 (1, 46)		.84
Second trial	–.044 (–0.381 to 0.468)		—		—
Third trial	[reference]				
Condition	—		2.993 (1, 48)		.09
Smoking agent	–.365 (–0.790 to 0.059)		—		—
Nonsmoking agent	[reference]				
iPQ^d^ score					
Age	–.012 (–0.128 to 0.103)		0.047 (1, 46)		.83
Prior abstinence	—		0.001 (1, 46)		.97
12 hours	–.048 (–2.600 to 2.505)		—		—
30 minutes	[reference]				
Sequence	—		4.6517 (1, 46)		.04^e^
Smoking agent first	2.634 (0.139 to 5.129)		—		—
Nonsmoking agent first	[reference]				
Time point	—		0.756 (1, 48)		.39
Second trial	–.261 (–0.342 to 0.863)		—		—
Third trial	[reference]				
Condition	—		0.023 (1, 48)		.88
Smoking agent	–.045 (–0.557 to 0.648)		—		—
Nonsmoking agent	[reference]				
Cortisol concentrations					
Age	–.010 (–0.009 to 0.029)		1.133 (1, 46)		.29
Prior abstinence	—		0.042 (1, 46)		.84
12 hours	–.042 (–0.374 to 0.459)		—		—
30 minutes	[reference]				
Sequence	—		0.022 (1, 46)		.88
Smoking agent first	–.030 (–0.437 to 0.377)		—		—
Nonsmoking agent first	[reference]				
Time point	—		18.463 (1, 48)		<.001^e^
Second trial	–.269 (0.143 to 0.396)		—		—
Third trial	[reference]				
Condition	—		0.307 (1, 48)		.58
Smoking agent	–.035 (–0.161 to 0.091)		—		—
Nonsmoking agent	[reference]				

a Cases n=50; Observations n=100.

b VAS: visual analog scale.

c Not applicable.

d iPQ: igroup Presence Questionnaire.

e *P*<.05

### Longitudinal Effect From Baseline to Marketplace With Interacting Agent to Follow-Up

**Figure 3.**
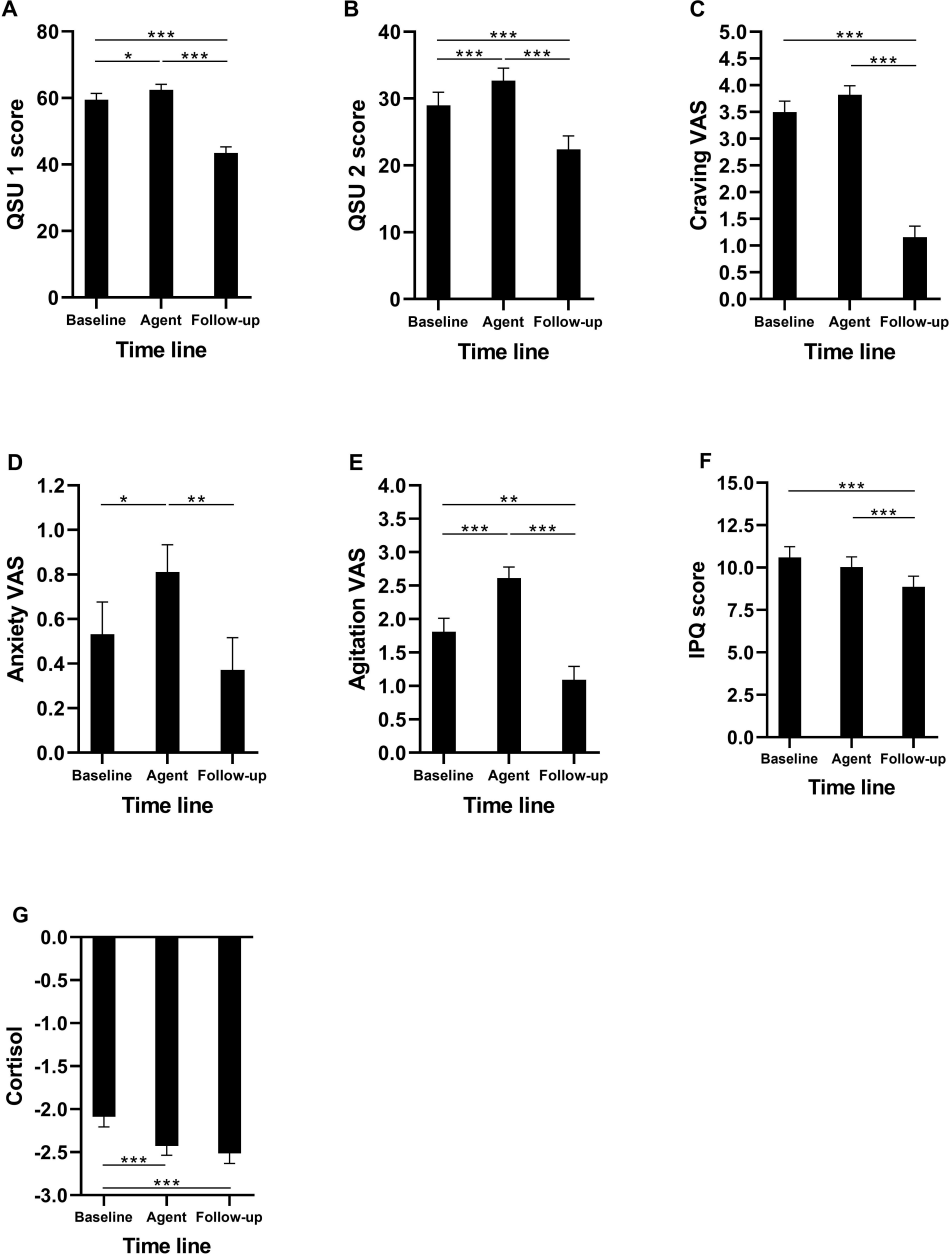
Bar graphs of estimated marginal means (+/– standard errors of the means) for QSU scales, VAS (craving, anxiety, agitation), iPQ sum scores, and salivary cortisol (logarithmized values) over the course of the experiment. The timeline is structured as baseline, agents (smoking|nonsmoking merged), and follow-up. Asterisks indicate significant differences between time points: * for *P*<.05, ** for *P*<.01, and *** for *P*<.001.

From baseline to the conditions with an agent present, there was an increase in craving in both QSU scales. For the craving VAS, the increase compared to the baseline was not significant. After smoking a cigarette, there was a reduction in craving in QSU and VAS. Anxiety and agitation increased from baseline to the agent conditions and decreased after smoking a cigarette. The iPQ score decreased from the baseline and agent conditions to the follow-up, and the cortisol concentrations reduced from baseline to the agent and follow-up conditions. Figure 3 depicts changes in the craving measures, anxiety, agitation, immersion, and salivary cortisol over the different conditions. Results tables are provided in Tables S2-S8 in Multimedia Appendix 1.

Participants who were abstinent for 12 hours reported overall higher cravings in VAS and QSU compared to participants who had been abstinent for 30 minutes. For salivary cortisol levels, immersion, anxiety, and agitation, the duration of prior abstinence was no significant predictor.

According to the qualitative grading system for iPQ scores by Melo and Gonçalves [33], the mean immersion scores were low for all three subscales. Over the course of the experiment, there was a decrease in immersion, with higher iPQ sum scores at baseline and lower scores at follow-up.

Salivary cortisol concentrations were highest at the beginning of the experiment and decreased from baseline to the agent and follow-up conditions. Follow-up cortisol levels did not differ significantly from the agent condition (Table S8 in Multimedia Appendix 1).

## Discussion

### Principal Findings

This study provides new insight into the interaction of contextual factors with proximal stimuli in relation to cigarette craving in a VR environment. Self-reported craving did not significantly differ in the presence of a smoking compared to a nonsmoking virtual agent. However, in the longitudinal design, the presence of an agent per se was associated with higher craving in the QSU compared to baseline, unrelated to the agent’s smoking status. As a positive control, smoking a cigarette was followed by a decrease in craving, which speaks for the validity of this study setup and highlights the applicability of the VAS within VR as a valid measure for explicit craving. The craving results were paralleled by the anxiety and agitation VAS, which were higher in the agent conditions compared to baseline and decreased after smoking a cigarette.

Our findings suggest that social context can induce craving independently of the presence of smoking-related cues. The presence of and interaction with a virtual agent was associated with more craving compared to baseline, but the agent smoking, who offered a cigarette, did not lead to a further increase in self-reported craving. Similarly, a VR study with patients with alcohol dependence [34] found that social pressure applied by virtual agents (offering drinks and trying to persuade participants to drink) without environmental alcohol cues induced a significant increase in craving. Social pressure with the addition of alcohol cues was not associated with more craving than social pressure on its own. While Lee et al [34] included agents pressuring participants to drink alcohol, the interaction in the nonsmoking agent condition did not reference the topic of smoking but rather referred to the participants as appearing quite stressed to the virtual agent. A possible explanation for this study’s results could be stimulus generalization [35]. When addictive substances like cigarettes and alcohol are often consumed in the presence of others, a social context that would normally be associated with substance use can elicit craving without the presence of specific substance-related cues. Studies with pictorial stimuli show that the combination of different drug-related stimuli does not necessarily have additive effects on cue-induced responses [36], which might explain why we did not find higher craving in the smoking compared to the nonsmoking agent condition.

This study’s findings differ from those of Winkler et al [8], who found that cue-induced craving in smokers was attenuated in the social context in a VR experiment. The authors provided the explanation that the presence of an agent who neither smokes nor applies social pressure to smoke might reduce the perceived availability of smoking. In contrast to our experiment, Winkler et al [8] did not include interactions with the virtual agents but had agents face the participants at a table with a neutral facial expression. It can be argued that the availability of smoking might be perceived as higher when the agent shows interest and interacts in a friendly way compared to noninteractive neutrality. Furthermore, the outdoor setting of our virtual environment might make smoking in the presence of another person, unrelated to their smoking status, appear less rude compared to the indoor setting of Winkler et al [8], which would also increase the perceived availability of smoking, and therefore, not lead to an attenuation of craving as argued by the authors. However, the smoking agent condition, which arguably had higher availability of smoking as the agent repeatedly offered a cigarette, did not lead to increased craving, which speaks against the perceived availability hypothesis. Generally, the discrepancy between our findings and those of Winkler et al [8] suggests that social context affects craving differently depending on the presence or absence of social interactions. Assessing how different aspects of social context might influence craving and modulate the response to smoking cues could be an interesting topic for further research.

We found an increase in craving from baseline to the agent conditions in the QSU, but not in the VAS. This is somewhat surprising, as the literature review shows high correlations between the QSU and craving VAS [37]. One possible explanation is that the VAS was administered within the VR, whereas the QSU was filled in immediately after each VR trial on a laptop, resulting in differences in the environment, but also the time participants had been exposed to the VR condition at the time of the two craving assessments. In addition, it can be argued that compared to a validated psychometric questionnaire like the QSU, the VAS assesses craving more broadly and may be less sensitive to changes in specific aspects of experienced craving.

Cortisol concentrations did not differ significantly between the smoking and nonsmoking agent conditions and decreased over the course of the experiment. Smoking a cigarette did not appear to have a significant effect on salivary cortisol concentration at follow-up. It is possible that participants experienced more stress at the beginning of the experiment due to the unfamiliar environment and experimental tasks, and decreased as they familiarized themselves with the VR environment over time. Considering the latency of poststress changes in salivary cortisol concentration [38], it is possible that potential cortisol peaks after cue exposure could not have been detected in our experiment due to the interval between cue exposure and saliva sampling being too short.

### Limitations

A limiting factor of this study is the weak immersion in the VR environment. The depth of immersion, as assessed with the iPQ, was classified as low and decreased over the course of the experiment. A study testing different VR environments for the induction of craving for cigarettes found a positive correlation between the subjective level of presence in the VR and craving [26]. In patients with alcohol use disorder, the perceived realism of alcoholic beverages in a VR environment is a predictor for craving [39]. It is possible that craving did not increase in the presence of the smoking agent because the smoking cues were not perceived as realistic enough. However, the same study found no correlation between the perceived realism of the overall VR environment and induced craving [39]. As participants only rated the overall immersion, but not the realism of specific aspects (eg, smoking cues) of the environment, it is unclear if the craving results were likely to be influenced by low immersion. Moreover, several studies indicate that while immersion is a relevant factor, its importance appears not to be central to study outcomes [40-42]. For this reason, we do not expect the low immersion to severely limit the validity of our results, albeit it certainly is a limiting factor.

Another limitation of this study is the lack of measures to evoke embodiment. Sense of embodiment in VR describes the sense of ownership, self-location, and agency over the virtual body [43] and is being discussed as one of the key factors that make VR applications effective, as it is suggested to mirror the brain mechanism of embodied simulations [44]. It has been shown to impact responses to virtual stimuli [45], and therefore, might influence cue-induced craving in VR. However, a study on VR gambling did not find an association between the level of embodiment and craving [46]. As ours was a small-budget pilot study, we were not able to implement measures to evoke embodiment, like programming a virtual avatar for participants or the inclusion of visuotactile stimulation. We also did not assess participants’ sense of embodiment. The role of embodiment in cue-induced craving in VR is a relevant topic for future research.

### Conclusions

In conclusion, this study shows that inducing cigarette craving in VR may not be as easy as some studies suggest. We were able to elicit craving with our VR environment, however, the presence of a smoking agent did not lead to the increase in craving we had expected when compared to a nonsmoking agent. This highlights the importance of manipulation checks in VR settings targeting craving and cue reactivity. According to our findings, social context in a virtual environment does have an effect on cigarette craving. However, which specific contextual factors are relevant or necessary for the induction and modulation of craving in smokers is not fully clear and requires further research. As VR allows the standardized presentation and isolated manipulation of social contextual stimuli, VR experiments present an exciting approach to address this question.

## Supplementary material

10.2196/71285 Multimedia Appendix 1 Supplementary materials.

## Abbreviations

iPQ: iGroup Presence Questionnaire
QSU: Questionnaire of Smoking Urges
VAS: visual analog scale
VR: virtual reality

## References

[1] Reitsma MB, Kendrick PJ, Ababneh E (2021). Spatial, temporal, and demographic patterns in prevalence of smoking tobacco use and attributable disease burden in 204 countries and territories, 1990–2019: a systematic analysis from the Global Burden of Disease Study 2019. Lancet.

[2] American Psychiatric Association (2013). Diagnostic and Statistical Manual of Mental Disorders.

[3] Conklin CA, Vella EJ, Joyce CJ, Salkeld RP, Perkins KA, Parzynski CS (2015). Examining the relationship between cue-induced craving and actual smoking. Exp Clin Psychopharmacol.

[4] Gass JC, Motschman CA, Tiffany ST (2014). The relationship between craving and tobacco use behavior in laboratory studies: a meta-analysis. Psychol Addict Behav.

[5] Vafaie N, Kober H (2022). Association of drug cues and craving with drug use and relapse. JAMA Psychiatry.

[6] Betts JM, Dowd AN, Forney M, Hetelekides E, Tiffany ST (2021). A meta-analysis of cue reactivity in tobacco cigarette smokers. Nicotine Tob Res.

[7] Vollstädt-Klein S, Nees F, Wieland A (2022). Contexts enhance ratings of craving and psychophysiological responses of cue-reactivity in tobacco use disorder. medRxiv.

[8] Winkler MH, Li Y, Pauli P, Mühlberger A (2023). Modulation of smoking cue reactivity by social context—implications for exposure therapy in virtual reality. Front Virtual Real.

[9] Dimoff JD, Sayette MA (2017). The case for investigating social context in laboratory studies of smoking. Addiction.

[10] Simons-Morton BG, Farhat T (2010). Recent findings on peer group influences on adolescent smoking. J Prim Prev.

[11] Hatsukami DK, Morgan SF, Pickens RW, Champagne SE (1990). Situational factors in cigarette smoking. Addict Behav.

[12] Shiffman S, Gwaltney CJ, Balabanis MH (2002). Immediate antecedents of cigarette smoking: an analysis from ecological momentary assessment. J Abnorm Psychol.

[13] Shiffman S, Paty JA, Gnys M, Kassel JA, Hickcox M (1996). First lapses to smoking: within-subjects analysis of real-time reports. J Consult Clin Psychol.

[14] Shiffman S, Rathbun SL (2011). Point process analyses of variations in smoking rate by setting, mood, gender, and dependence. Psychol Addict Behav.

[15] Childs E, de Wit H (2010). Effects of acute psychosocial stress on cigarette craving and smoking. Nicotine Tob Res.

[16] Buchmann AF, Laucht M, Schmid B, Wiedemann K, Mann K, Zimmermann US (2010). Cigarette craving increases after a psychosocial stress test and is related to cortisol stress response but not to dependence scores in daily smokers. J Psychopharmacol.

[17] Wanger TJ, de Moura FB, Ashare R (2023). Brain and cortisol responses to smoking cues are linked in tobacco-smoking individuals. Addict Biol.

[18] Pan X, Hamilton AF de C (2018). Why and how to use virtual reality to study human social interaction: the challenges of exploring a new research landscape. Br J Psychol.

[19] Yaremych HE, Persky S (2019). Tracing physical behavior in virtual reality: a narrative review of applications to social psychology. J Exp Soc Psychol.

[20] Thompson-Lake DGY, Cooper KN, Mahoney JJ (2015). Withdrawal symptoms and nicotine dependence severity predict virtual reality craving in cigarette-deprived smokers. Nicotine Tob Res.

[21] Choi JS, Park S, Lee JY (2011). The effect of repeated virtual nicotine cue exposure therapy on the psychophysiological responses: a preliminary study. Psychiatry Investig.

[22] Lee JH, Ku J, Kim K (2003). Experimental application of virtual reality for nicotine craving through cue exposure. CyberPsychol Behav.

[23] Mazza M, Kammler-Sücker K, Leménager T, Kiefer F, Lenz B (2021). Virtual reality: a powerful technology to provide novel insight into treatment mechanisms of addiction. Transl Psychiatry.

[24] Park CB, Choi JS, Park SM (2014). Comparison of the effectiveness of virtual cue exposure therapy and cognitive behavioral therapy for nicotine dependence. Cyberpsychol Behav Soc Netw.

[25] Moon J, Lee JH (2009). Cue exposure treatment in a virtual environment to reduce nicotine craving: a functional MRI study. Cyberpsychol Behav.

[26] Ferrer-García M, García-Rodríguez O, Gutiérrez-Maldonado J, Pericot-Valverde I, Secades-Villa R (2010). Efficacy of virtual reality in triggering the craving to smoke: its relation to level of presence and nicotine dependence. Stud Health Technol Inform.

[27] Hoffmann S, Gerhardt S, Mühle C (2024). Associations of menstrual cycle and progesterone-to-estradiol ratio with alcohol consumption in alcohol use disorder: a sex-separated multicenter longitudinal study. Am J Psychiatry.

[28] Heatherton TF, Kozlowski LT, Frecker RC, Fagerström KO (1991). The Fagerström Test for Nicotine Dependence: a revision of the Fagerström Tolerance Questionnaire. Br J Addict.

[29] Klein EM, Brähler E, Dreier M (2016). The German version of the Perceived Stress Scale—psychometric characteristics in a representative German community sample. BMC Psychiatry.

[30] Tiffany ST, Drobes DJ (1991). The development and initial validation of a questionnaire on smoking urges. Br J Addict.

[31] Schubert TW (2003). [Experience of presence in virtual exercises: a scale for measuring spatial presence, involvement, and reality judgment]. Z Medienpsychol.

[32] Kennedy RS, Lane NE, Berbaum KS, Lilienthal MG (1993). Simulator Sickness Questionnaire: an enhanced method for quantifying simulator sickness. Int J Aviat Psychol.

[33] Melo M, Gonçalves G, Vasconcelos-Raposo josé, Bessa M (2023). How much presence is enough? qualitative scales for interpreting the Igroup Presence Questionnaire score. IEEE Access.

[34] Lee JS, Namkoong K, Ku J (2008). Social pressure-induced craving in patients with alcohol dependence: application of virtual reality to coping skill training. Psychiatry Investig.

[35] Andreatta M, Pauli P (2019). Generalization of appetitive conditioned responses. Psychophysiology.

[36] Mucha RF, Pauli P, Weber M, Winkler M (2008). Smoking stimuli from the terminal phase of cigarette consumption may not be cues for smoking in healthy smokers. Psychopharmacology (Berl).

[37] Acquadro C, Desvignes-Gleizes C, Mainy N, Hankins M, Weitkunat R, Chrea C (2019). Measurement properties of the translations of instruments evaluating the subjective effects of tobacco- and nicotine-containing products: a systematic review of the literature. F1000Res.

[38] Lopez-Duran NL, Mayer SE, Abelson JL (2014). Modeling neuroendocrine stress reactivity in salivary cortisol: adjusting for peak latency variability. Stress.

[39] Hernández-Serrano O, Ghiţă A, Fernández-Ruiz J (2021). Determinants of cue-elicited alcohol craving and perceived realism in virtual reality environments among patients with alcohol use disorder. J Clin Med.

[40] Kovacs M, Campos F, Vugts V (2019). Augmented Reality and Virtual Reality: The Power of AR and VR for Business.

[41] Bowman DA, McMahan RP (2007). Virtual reality: how much immersion is enough?. Computer (Long Beach Calif).

[42] Rose T, Nam CS, Chen KB (2018). Immersion of virtual reality for rehabilitation—review. Appl Ergon.

[43] Kilteni K, Groten R, Slater M (2012). The sense of embodiment in virtual reality. Presence: Teleoperators Virtual Environ.

[44] Riva G, Wiederhold BK, Mantovani F (2019). Neuroscience of virtual reality: from virtual exposure to embodied medicine. Cyberpsychol Behav Soc Netw.

[45] Gall D, Roth D, Stauffert JP, Zarges J, Latoschik ME (2021). Embodiment in virtual reality intensifies emotional responses to virtual stimuli. Front Psychol.

[46] Oberdörfer S, Schraudt D, Latoschik ME (2022). Embodied gambling—investigating the influence of level of embodiment, avatar appearance, and virtual environment design on an online VR slot machine. Front Virtual Real.

