# Supplementary material for: Reactivity to Smoking Cues in a Social Context: Virtual Reality Experiment
Source: JMIR Form Res. 2025 May 26;9:e71285. doi: 10.2196/71285 (PMC12129368; doi:10.2196/71285)
Supplement: Multimedia Appendix 1 [file formative-v9-e71285-s001.docx]

Table S1. Results of a multilevel model with the craving VAS as the dependent variable and age, duration of prior abstinence, sequence, time point (trial 2 or 3), condition and the interaction term of duration of prior abstinence and time point as predictors.

|  | β | 95% CI | F | df1, df2 | p |
| --- | --- | --- | --- | --- | --- |
| N[cases] = 50  N[observations] = 100 |  |  |  |  |  |
| Age | -0.008 | [-0.044, 0.028] | 0.190 | (1, 46) | 0.665 |
| Prior Abstinence |  |  | 6.699 | (1, 46) | 0.013 |
| 12 hours | 1,017 | [0.226, 1.809] |  |  |  |
| 30 Minutes | [Reference] |  |  |  |  |
| Sequence |  |  | 0.308 | (1, 46) | 0.582 |
| Smoking Agent First | -0.213 | [-0.987, 0.560] |  |  |  |
| Non-smoking Agent First | [Reference] |  |  |  |  |
| Time point |  |  | 4.384 | (1, 48) | 0.042 |
| Second trial | -0.451 | [-0.885, 0.018] |  |  |  |
| Third trial | [Reference] |  |  |  |  |
| Condition |  |  | 0.191 | (1, 48) | 0.664 |
| Smoking Agent | 0.094 | [-0.339, 0.528] |  |  |  |
| Non-smoking Agent | [Reference] |  |  |  |  |
| Time point x Prior abstinence | 0.223 | [-0.666, 1.109] | 0.253 | (1, 47) | 0.618 |

Table S2. Results of a multilevel model with craving VAS as the dependent variable and age, prior abstinence, sequence and condition (baseline, agent present, follow up) as predictors.

|  | β | 95% CI | t | p | F | df1, df2 | p |
| --- | --- | --- | --- | --- | --- | --- | --- |
| N[cases] = 50  N[observations] = 191 |  |  |  |  |  |  |  |
| Age | -0.007 | [-0.035, 0.020] |  |  | 0.304 | (1, 46) | 0.584 |
| Prior Abstinence |  |  |  |  | 11.930 | (1, 46) | **0.001** |
| 12 hours | 1.035 | [0.432, 1.639] |  |  |  |  |  |
| 30 Minutes | [Reference] |  |  |  |  |  |  |
| Sequence |  |  |  |  | 4.076 | (1, 46) | **0.049** |
| Smoking Agent First | -0.592 | [-1.181, -0.002] |  |  |  |  |  |
| Non-smoking Agent First | [Reference] |  |  |  |  |  |  |
| Condition |  |  |  |  | 93.902 | (2, 148) | **<0.001** |
| Baseline | -0.320 | [-0.713, 0.073] | -1.608 | 0.110 |  |  |  |
| Smoking Agent/ Non-smoking Agent | [Reference] |  |  |  |  |  |  |
| Follow Up | -2.660 | [-3.053, -2.267] | -13.367 | **<0.001** |  |  |  |
| The multilevel models include fixed and random intercepts. | | | | | | | |

Table S3. Results of a multilevel model with QSU factor 1 as the dependent variable and age, prior abstinence, sequence and condition (baseline, agent present, follow up) as predictors.

|  | β | 95% CI | t | p | F | df1, df2 | p |
| --- | --- | --- | --- | --- | --- | --- | --- |
| N[cases] = 50  N[observations] = 191 |  |  |  |  |  |  |  |
| Age | 0.231 | [-0.060, 0.521] |  |  | 2.549 | (1, 46) | 0.117 |
| Prior Abstinence |  |  |  |  | 11.202 | (1, 46) | **0.002** |
| 12 hours | 10.717 | [4.272, 17.161] |  |  |  |  |  |
| 30 Minutes | [Reference] |  |  |  |  |  |  |
| Sequence |  |  |  |  | 1.033 | (1, 46) | 0.315 |
| Smoking Agent First | -3.176 | [-9.465, 3.113] |  |  |  |  |  |
| Non-smoking Agent First | [Reference] |  |  |  |  |  |  |
| Condition |  |  |  |  | 100.417 | (2, 140) | **<0.001** |
| Baseline | -2.954 | [-5.660, -0.248] | -2.158 | **0.033** |  |  |  |
| Smoking Agent/ Non-smoking Agent | [Reference] |  |  |  |  |  |  |
| Follow Up | -18.970 | [-21.671, -16.269] | -13.887 | **<0.001** |  |  |  |
| The multilevel models include fixed and random intercepts. | | | | | | | |

Table S4. Results of a multilevel model with QSU factor 2 as the dependent variable and age, prior abstinence, sequence and condition (baseline, agent present, follow up) as predictors.

|  | β | 95% CI | t | p | F | df1, df2 | p |
| --- | --- | --- | --- | --- | --- | --- | --- |
| N[cases] = 50  N[observations] = 196 |  |  |  |  |  |  |  |
| Age | 0.191 | [-0.146, 0.528] |  |  | 1.298 | (1, 46) | 0.260 |
| Prior Abstinence |  |  |  |  | 5.549 | (1, 46) | **0.023** |
| 12 hours | 8.691 | [1.264, 16.119] |  |  |  |  |  |
| 30 Minutes | [Reference] |  |  |  |  |  |  |
| Sequence |  |  |  |  | 0.002 | (1, 46) | 0.961 |
| Smoking Agent First | -0.175 | [-7.435, 7.085] |  |  |  |  |  |
| Non-smoking Agent First | [Reference] |  |  |  |  |  |  |
| Condition |  |  |  |  | 43.814 | (2, 144) | **<0.001** |
| Baseline | -3.707 | [-5.815, -1.598] | -3.475 | **<0.001** |  |  |  |
| Smoking Agent/ Non-smoking Agent | [Reference] |  |  |  |  |  |  |
| Follow Up | -10.250 | [-12.416, -8.083] | -9.351 | **<0.001** |  |  |  |
| The multilevel models include fixed and random intercepts. | | | | | | | |

Table S5. Results of a multilevel model with anxiety VAS as the dependent variable and age, prior abstinence, sequence and condition (baseline, agent present, follow up) as predictors.

|  | β | 95% CI | t | p | F | df1, df2 | p |
| --- | --- | --- | --- | --- | --- | --- | --- |
| N[cases] = 50  N[observations] = 191 |  |  |  |  |  |  |  |
| Age | -0.000 | [-0.020, 0.019] |  |  | 0.002 | (1, 46) | 0.964 |
| Prior Abstinence |  |  |  |  | 0.704 | (1, 46) | 0.406 |
| 12 hours | -0.183 | [-0.620, 0.255] |  |  |  |  |  |
| 30 Minutes | [Reference] |  |  |  |  |  |  |
| Sequence |  |  |  |  | 0.589 | (1, 46) | 0.447 |
| Smoking Agent First | -0.163 | [-0.591, 0.264] |  |  |  |  |  |
| Non-smoking Agent First | [Reference] |  |  |  |  |  |  |
| Condition |  |  |  |  | 5.642 | (2, 148) | **0.004** |
| Baseline | -2.80 | [-0.552, -0.008] | -2.035 | **0.044** |  |  |  |
| Smoking Agent/ Non-smoking Agent | [Reference] |  |  |  |  |  |  |
| Follow Up | -0.440 | [-0.712, -0.168] | -3.198 | **0.002** |  |  |  |
| The multilevel models include fixed and random intercepts. | | | | | | | |

Table S6. Results of a multilevel model with agitation VAS as the dependent variable and age, prior abstinence, sequence and condition (baseline, agent present, follow up) as predictors

|  | β | 95% CI | t | p | F | df1, df2 | p |
| --- | --- | --- | --- | --- | --- | --- | --- |
| N[cases] = 50  N[observations] = 191 |  |  |  |  |  |  |  |
| Age | -0.017 | [-0.043, 0.010] |  |  | 1.579 | (1, 46) | 0.215 |
| Prior Abstinence |  |  |  |  | 0.237 | (1, 46) | 0.629 |
| 12 hours | 0.142 | [-0.447, 0.732] |  |  |  |  |  |
| 30 Minutes | [Reference] |  |  |  |  |  |  |
| Sequence |  |  |  |  | 1.058 | (1, 46) | 0.309 |
| Smoking Agent First | -0.294 | [-0.870, 0.282] |  |  |  |  |  |
| Non-smoking Agent First | [Reference] |  |  |  |  |  |  |
| Condition |  |  |  |  | 31.540 | (2, 148) | **<0.001** |
| Baseline | -0.800 | [-1.186, -0.414] | -4.095 | **<0.001** |  |  |  |
| Smoking Agent/ Non-smoking Agent | [Reference] |  |  |  |  |  |  |
| Follow Up | -1.520 | [-1.906, -1.134] | -7.781 | **<0.001** |  |  |  |
| The multilevel models include fixed and random intercepts. | | | | | | | |

Table S7. Results of a multilevel model with the iPQ sum score as the dependent variable and age, prior abstinence, sequence and condition (baseline, agent present, follow-up) as predictors.

|  | β | 95% CI | t | p | F | df1, df2 | p |
| --- | --- | --- | --- | --- | --- | --- | --- |
| N[cases] = 50  N[observations] = 199 |  |  |  |  |  |  |  |
| Age | -0.009 | [-0.117, 0.098] |  |  | 0.030 | (1, 46) | 0.864 |
| Prior Abstinence |  |  |  |  | 0.078 | (1, 46) | 0.781 |
| 12 hours | -0.329 | [-2.703, 2.044] |  |  |  |  |  |
| 30 Minutes | [Reference] |  |  |  |  |  |  |
| Sequence |  |  |  |  | 4.280 | (1, 46) | **0.044** |
| Smoking Agent First | 2.385 | [0.065, 4.705] |  |  |  |  |  |
| Non-smoking Agent First | [Reference] |  |  |  |  |  |  |
| Condition |  |  |  |  | 11.666 | (2, 147) | **<0.001** |
| Baseline | 0.576 | [-0.060, 1.212] | 1.790 | 0.075 |  |  |  |
| Smoking Agent/ Non-smoking Agent | [Reference] |  |  |  |  |  |  |
| Follow Up | -1.162 | [-1.792, 0.532] | -3.643 | **<0.001** |  |  |  |
| The multilevel models include fixed and random intercepts. | | | | | | | |

Table S8. Results of a multilevel model with cortisol level as the dependent variable and age, prior abstinence, sequence and condition (baseline, agent present, follow up) as predictors.

|  | β | 95% CI | t | p | F | df1, df2 | p |
| --- | --- | --- | --- | --- | --- | --- | --- |
| N[cases] = 50  N[observations] = 200 |  |  |  |  |  |  |  |
| Age | 0.008 | [-0.011, 0.027] |  |  | 0.649 | (1, 46) | 0.424 |
| Prior Abstinence |  |  |  |  | 0.023 | (1, 46) | 0.879 |
| 12 hours | 0.032 | [-0.387, 0.450] |  |  |  |  |  |
| 30 Minutes | [Reference] |  |  |  |  |  |  |
| Sequence |  |  |  |  | 0.058 | (1, 46) | 0.811 |
| Smoking Agent First | 0.049 | [-0.360, 0.458] |  |  |  |  |  |
| Non-smoking Agent First | [Reference] |  |  |  |  |  |  |
| Treat |  |  |  |  | 12.770 | (2, 148) | **<0.001** |
| Baseline | 0.339 | [0.183, 0.494] | 4.293 | **<0.001** |  |  |  |
| Smoking Agent/ Non-smoking Agent | [Reference] |  |  |  |  |  |  |
| Follow Up | -0.085 | [-0.241, 0.070] | -1.083 | 0.281 |  |  |  |
| The multilevel models include fixed and random intercepts. | | | | | | | |
